# Inferring locomotor behaviours in Miocene New World monkeys using finite element analysis, geometric morphometrics and machine-learning classification techniques applied to talar morphology

**DOI:** 10.1098/rsif.2018.0520

**Published:** 2018-09-26

**Authors:** Thomas A. Püschel, Jordi Marcé-Nogué, Justin T. Gladman, René Bobe, William I. Sellers

**Affiliations:** 1 School of Earth and Environmental Sciences, University of Manchester, Manchester M13 9PL, UK; 2 Center of Natural History (CeNak), Universität Hamburg, Martin-Luther-King-Platz 3, Hamburg 20146, Germany; 3 Institut Català de Paleontologia M. Crusafont, Universitat Autònoma de Barcelona, Cerdanyola del Vallès, Barcelona 08193, Spain; 4 Department of Engineering, Shared Materials Instrumentation Facility (SMIF), Duke University, Durham, NC, USA; 5 Departamento de Antropología, Universidad de Chile, Santiago, Chile; 6 Institute of Cognitive and Evolutionary Anthropology, School of Anthropology, University of Oxford, Oxford, UK

**Keywords:** Platyrrhini, finite-element modelling, morphometrics, talus, statistical learning, positional behaviour

## Abstract

The talus is one of the most commonly preserved post-cranial elements in the platyrrhine fossil record. Talar morphology can provide information about postural adaptations because it is the anatomical structure responsible for transmitting body mass forces from the leg to the foot. The aim of this study is to test whether the locomotor behaviour of fossil Miocene platyrrhines could be inferred from their talus morphology. The extant sample was classified into three different locomotor categories and then talar strength was compared using finite-element analysis. Geometric morphometrics were used to quantify talar shape and to assess its association with biomechanical strength. Finally, several machine-learning (ML) algorithms were trained using both the biomechanical and morphometric data from the extant taxa to infer the possible locomotor behaviour of the Miocene fossil sample. The obtained results show that the different locomotor categories are distinguishable using either biomechanical or morphometric data. The ML algorithms categorized most of the fossil sample as arboreal quadrupeds. This study has shown that a combined approach can contribute to the understanding of platyrrhine talar morphology and its relationship with locomotion. This approach is likely to be beneficial for determining the locomotor habits in other fossil taxa.

## 1. Introduction

**Figure 1.**
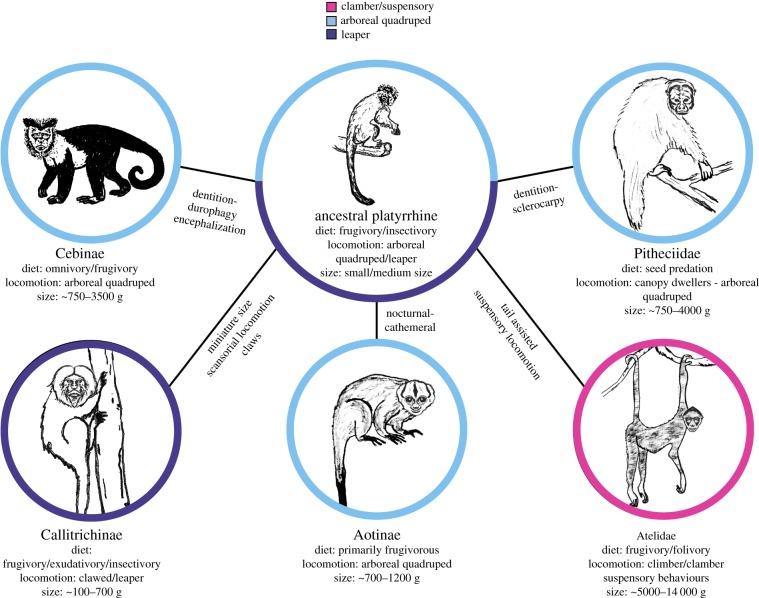
Broad platyrrhine ecophyletic groups. Colours represent different main locomotion modes. (Online version in colour.)

Extant platyrrhines or New World monkeys (NWM) inhabit a diverse range of habitats in the Americas [1]. The occupation of these niches has been coupled by distinct behavioural, locomotor, morphological and ecological adaptations in each one of the main platyrrhine clades [2], which can be summarized in broad ecophyletic groups (figure 1). One of the main difficulties in NWM palaeobiology is the scarceness of fossils from the Eocene and Oligocene, with most NWM fossils dated to the Miocene or the Pleistocene of the Caribbean and South America [3], although it is important to note that there have been outstanding but rare findings in Bolivia and Peru [4]. Even though the fossil record of NWM has notably improved over the last decade, it is particularly intriguing that the majority of the NWM fossil record for the Early Miocene has been found in middle and high latitudes (i.e. central Chile and Patagonia), which are no longer areas occupied by any extant platyrrhine [5].

After teeth, the talus is probably the most commonly preserved anatomical element in the platyrrhine fossil record [3], with several Miocene taxa possessing at least one conserved talus [6]. Importantly, talar morphology can provide insights about postural adaptations due to its interconnection with other foot bones [7,8]. The talus is also the principal mechanical connection between the leg and the foot and is responsible for transmitting body weight, as well as providing stability and mobility throughout locomotor behaviours [7]. The combination of its high occurrence and good preservation in the fossil record and its functional role in the ankle joint make it a valuable element when hypothesizing the postural and locomotor behaviours of fossil primates [9,10].

There is a strong and significant association between talar shape and locomotor behaviour [6], and evidence shows that bone is functionally adapted to the mechanical demands that are imposed during life [11]. Therefore, it is logical to hypothesize that talar mechanical strength associated with biomechanical performance could also be used to distinguish and infer locomotor behaviours. Currently, there is an absence of comparative biomechanical analyses that could provide important information about the usefulness of talar biomechanical performance as a positional behaviour proxy [12]. Consequently, we analysed the biomechanical performance of the extant platyrrhine talar morphological diversity by applying finite-element analysis (FEA). There is an almost total absence of studies applying FEA to primate, let alone platyrrhine, talar biomechanics. To our knowledge, most studies analysing primate talar biomechanics using FEA have focused on human feet (e.g. [13–15]). Thus, the present contribution represents an important step in analysing an extensive non-human primate comparative sample using FEA. Since we were also interested in the relationship between talar biomechanical performance and its morphology, we used geometric morphometrics (GMs) to collect shape data. In addition, because our objective was to classify the fossils into different locomotor categories, several machine-learning (ML) algorithms were trained using the extant biomechanical data to infer the locomotor categories of the Miocene fossil sample. Traditionally, most morphometric and also some of the FEA output analyses have been performed with reference to simple linear models [16,17]. For instance, when dealing with classification problems, most publications rely on linear discriminant analyses (or its more general extension, canonical variate analyses), in spite of the known limitations of these approaches [18,19]. Although the application of ML algorithms to tackle problems of specimen identification or group characterization has a vast literature in other biological fields [20], only more recently have several ML methods been applied using morphometric or biomechanical data (e.g. [13–15,21–26]). In addition, most of them have not compared different algorithms applied to the same problem. Therefore, some of these ML procedures were explored and their classification accuracy was assessed when applied to the problem of classifying our Miocene fossil sample using morphometric and biomechanical data.

Consequently, this study had three main aims for which we employed three different approaches. (i) The first goal was to test if there were significant differences in talar strength depending on locomotor categories to assess if different locomotor groups exhibit or not differences in biomechanical performance. Therefore, we classified our extant sample into broad locomotor categories and investigated whether there were dissimilar biomechanical performances depending on the locomotor category by simulating a static loading case using FEA. (ii) The second aim was to evaluate if there was an association between talar shape and stress data to test if shape covaries or not with biomechanical performance. Hence, we collected talar morphometric data to evaluate if there was an association between these two kinds of data by using partial least-squares analysis (PLS). (iii) Finally, our main goal was to classify the Miocene fossil sample into locomotor categories to infer broad locomotor behaviours. Therefore, several ML algorithms were trained and tested using the biomechanical and morphometric data and used to infer the possible locomotor behaviour of the extinct specimens.

## 2. Material and methods

### 2.1. Sample

The extant NWM sample included one talus from nearly every modern platyrrhine genus (40 species; table 1), whereas the fossil sample considered one talus from most of the available Miocene platyrrhine tali (10 specimens; table 2). The extant platyrrhine species were classified according to their main mode of locomotion in three categories (i.e. clamber/suspensory, leaper and arboreal quadruped) based on the locomotor mode percentages compiled by Youlatos & Meldrum [28] to compare if there were differences due to different locomotor modes (table 1).

**Table 1.** Extant sample.

species	subfamily	locomotion	sex	average body mass (g)^a^	accession number	museum/database
*Alouatta caraya*	Alouattinae	clamber/suspensory	male	5375	AMNH211513	Morphosource (http://morphosource.org/)
*Alouatta seniculus*	Alouattinae	clamber/suspensory	male	5950	AMNH23549	American Museum of Natural History
*Aotus azarae*	Cebinae	arboreal quadruped	male	1205	AMNH211458	American Museum of Natural History
*Aotus infulatus*	Cebinae	arboreal quadruped	female	1215	AMNH94992	Morphosource (http://morphosource.org/)
*Aotus nancymaae*	Cebinae	arboreal quadruped	male	787	AMNH239851	Morphosource (http://morphosource.org/)
*Aotus trivirgatus*	Cebinae	arboreal quadruped	female	786	AMNH187963	American Museum of Natural History
*Ateles belzebul*	Atelinae	clamber/suspensory	male	8070	AMNH95040	American Museum of Natural History
*Ateles fusciceps*	Atelinae	clamber/suspensory	male	9025	AMNH188140	Morphosource (http://morphosource.org/)
*Ateles geoffroyi*	Atelinae	clamber/suspensory	male	7535	AMNH28420	American Museum of Natural History
*Ateles marginatus*	Atelinae	clamber/suspensory	male	10230	AMNH95040	American Museum of Natural History
*Cacajao calvus*	Pitheciinae	arboreal quadruped	male	3165	USNM319516	National Museum of Natural History; Smithsonian Institution
*Callicebus cupreus*	Callicebinae	arboreal quadruped	male	1070	AMNH136208	American Museum of Natural History
*Callicebus donacophilus*	Callicebinae	arboreal quadruped	male	950	AMNH211487	American Museum of Natural History
*Callicebus moloch*	Callicebinae	arboreal quadruped	unknown	988	AMNH210393	Morphosource (http://morphosource.org/)
*Callicebus personatus*	Callicebinae	arboreal quadruped	female	1325	USNM240088	National Museum of Natural History; Smithsonian Institution
*Callicebus torquatus*	Callicebinae	arboreal quadruped	female	1325	USNM398212	National Museum of Natural History; Smithsonian Institution
*Callimico goeldii*	Callithrichinae	leaper	male	483.5	USNM395455	National Museum of Natural History; Smithsonian Institution
*Callithrix geoffroyi*	Callithrichinae	leaper	male	359	USNM582900	National Museum of Natural History; Smithsonian Institution
*Callithrix jacchus*	Callithrichinae	leaper	male	320.5	USNM399034	National Museum of Natural History; Smithsonian Institution
*Callithrix penicillata*	Callithrichinae	leaper	female	325.5	AMNH133692	American Museum of Natural History
*Cebuella pygmaea*	Callithrichinae	leaper	male	116	USNM303037	National Museum of Natural History; Smithsonian Institution
*Cebus albifrons*	Cebinae	arboreal quadruped	male	2735	AMNH209924	American Museum of Natural History
*Cebus apella*	Cebinae	arboreal quadruped	male	3085	AMNH133607	American Museum of Natural History
*Cebus nigritus*	Cebinae	arboreal quadruped	male	2825	USNM518478	National Museum of Natural History; Smithsonian Institution
*Cebus olivaceus*	Cebinae	arboreal quadruped	male	2905	AMNH30197	American Museum of Natural History
*Chiropotes satanas*	Pitheciinae	arboreal quadruped	male	2740	AMNH95760	Morphosource (http://morphosource.org/)
*Lagothrix lagotricha*	Atelinae	clamber/suspensory	male	7150	AMNH188153	American Museum of Natural History
*Leontopithecus rosalia*	Callithrichinae	leaper	male	609	USNM588152	National Museum of Natural History; Smithsonian Institution
*Mico argentatus*	Callithrichinae	leaper	male	345	USNM399069	National Museum of Natural History; Smithsonian Institution
*Mico humeralifer*	Callithrichinae	leaper	unknown	473.5	AMNH188164	American Museum of Natural History
*Mico melanurus*	Callithrichinae	leaper	female	350	USNM574137	National Museum of Natural History; Smithsonian Institution
*Pithecia monachus*	Pitheciinae	arboreal quadruped	male	2360	USNM395692	National Museum of Natural History; Smithsonian Institution
*Pithecia pithecia*	Pitheciinae	arboreal quadruped	male	1760	AMNH149149	Morphosource (http://morphosource.org/)
*Saguinus fuscicolis*	Callithrichinae	leaper	unknown	350.5	AMNH147433	American Museum of Natural History
*Saguinus leucopus*	Callithrichinae	leaper	female	492	AMNH148322	American Museum of Natural History
*Saguinus midas*	Callithrichinae	leaper	male	545	AMNH97316	Morphosource (http://morphosource.org/)
*Saguinus mystax*	Callithrichinae	leaper	male	524.5	AMNH188171	Morphosource (http://morphosource.org/)
*Saguinus oedipus*	Callithrichinae	leaper	female	411	AMNH200882	American Museum of Natural History
*Saimiri boliviensis*	Cebinae	arboreal quadruped	male	811	AMNH211596	American Museum of Natural History
*Saimiri sciureus*	Cebinae	arboreal quadruped	male	720.5	AMNH188090	Morphosource (http://morphosource.org/)

^a^Body mass data from Smith & Jungers [27].

**Table 2.** Fossil sample.

fossil	age	locality	body mass estimates (g)^a^	accession number	museum
*Dolichocebus gaimanensis*	approximately 20.0 Ma	Sarmiento, Chubut, Argentina	1601	MACN 362	Museo Argentino de Ciencias Naturales ‘Bernardino Rivadavia’, Buenos Aires, Argentina
*Carlocebus carmenensis*	17.5–16.5 Ma	Pinturas, Santa Cruz, Argentina	2914	MACN304	Museo Argentino de Ciencias Naturales ‘Bernardino Rivadavia’, Buenos Aires, Argentina
*Soriacebus ameghinorum*	17.5–16.5 Ma	Pinturas, Santa Cruz, Argentina	1721	MACN 397	Museo Argentino de Ciencias Naturales ‘Bernardino Rivadavia’, Buenos Aires, Argentina
Madre de Dios^b^	approximately 18.8–16.5 Ma	Atalaya, Cusco, Upper Madre de Dios Basin, Peru	352	MUSM 2024	Museo de Historia Natural de la Universidad Nacional Mayor San Marcos, Lima, Peru
Río Cisnes^b^	16.5 Ma	Alto Río Cisnes, Chile	1510	SGO.PV 974	Museo Nacional de Historia Natural, Santiago, Chile
*Proteropithecia neuquenensis*	15.8 Ma	Collón Curá, Neuquén, Argentina	2006	MLP 91-IX-1–119	Museo de La Plata, La Plata, Argentina
*Aotus dindensis*^c^	13.2–13 Ma	La Venta, Madgalena Valley, Colombia	874	IGMKU 8802	Museo Geológico, INGEOMINAS, Bogotá, Colombia
*Cebupithecia sarmientoi*	13.5–11.8 Ma	La Venta, Madgalena Valley, Colombia	1825	UCMP 38762	University of California, Berkeley Museum of Paleontology, Berkeley, California, USA
*Neosaimiri fieldsi*^c^	13.2–12 Ma	La Venta, Madgalena Valley, Colombia	781	IGMKU 89031	Museo Geológico, INGEOMINAS, Bogotá, Colombia
*Paralouatta marianae*^b^	approximately 18.5–17.5 Ma	Domo de Zaza, Lagunitas Formation, Cuba	4709	MNHNCu 76.3059	Museo Nacional de Historia Natural de Cuba, La Habana, Cuba

^a^Body mass estimates from Püschel *et al.* [6].

^b^Specimens that have not been taxonomically assigned.

^c^Scans obtained from casts.

#### 2.1.1. Phylogeny

A platyrrhine phylogeny [29] was slightly modified to include some species that were initially not present and to remove others that were in the phylogeny but for which there were no talar data. This phylogeny was used when carrying out the described comparative analyses and is available in the electronic supplementary material, file S1.

### 2.2. Three-dimensional model rendering

Surface models were imported into Geomagic Studio^®^ (3D Systems, v. 12, Rock Hill, SC, USA), where irregularities from scanning were repaired using refinement and smoothing tools. The tali were aligned according to the standard anatomical position (further details about the alignment procedure can be found in the electronic supplementary material, document S2.1). Some of the analysed fossils (i.e. *Dolichocebus*, *Soriacebus* and Río Cisnes) exhibit damage due to post-depositional processes. Their missing anatomical regions were virtually reconstructed to generate models suitable for FEA, so particular attention is required when interpreting their results. The case-specific reconstruction methods that were applied are described in electronic supplementary material, document S2.2.

### 2.3. Finite-element analysis

The models of the tali were imported into ANSYS^®^ (Ansys, Inc., v. 17.1, Canonsburg, PA, USA; http://www.ansys.com/) to perform the FEA modelling. The tali were modelled as solids composed only of cortical bone to simplify the analyses and to limit the number of assumptions. Homogeneous, linear and elastic material properties were assumed for the talar models. Cortical bone values from a human talus were used (Young's modulus: 20.7 GPa; Poisson's ratio: 0.3) [30]. The models were meshed with an adaptive mesh of hexahedral elements [31] meeting the conditions defined in [32] to create a Quasi-Ideal Mesh (QIM). Further information about the FEA models along with all of their results can be found in electronic supplementary material, table S3.

#### 2.3.1. Loading scenario and boundary conditions

**Figure 2.**
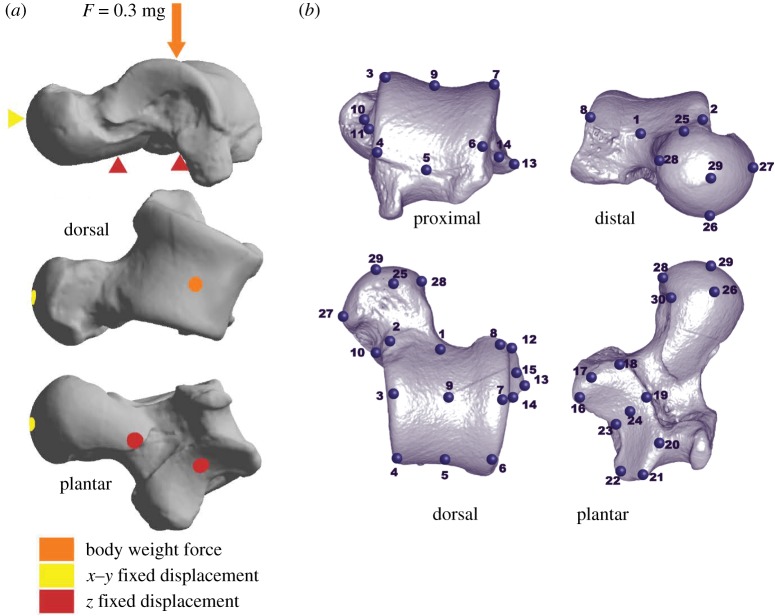
(*a*) Loading scenario tested in the FEA; (*b*) the 30 landmarks used in the GM analyses. (Online version in colour.)

Extant body mass data were obtained from Smith & Jungers [27], while the fossil body mass predictions were obtained from Püschel *et al.* [6]. Among living platyrrhine species, male and female body mass are highly correlated [29]; therefore, average body mass was used in the subsequent analyses (tables 1 and 2). Based on this information, we computed a value we called ‘body weight force’, which represents the applied load that was defined as the 30% of the average body mass of each species multiplied by gravitational acceleration *g* = 9.81 ms^−2^. This load was applied on the trochlear surface of each talus, thus simulating a basic quadrupedal scenario (in most monkeys, the hind limbs support more weight, hence the decision to apply 30% of the average body mass [33]). This load was directed in the direction of the *z*-axis on the oriented tali to simulate the action of gravity and was located at the centre of the trochlear surface to simulate a compressive force. The talus was constrained on the area comprising the sub-talar joint as indicated in figure 2*a*. In addition, a multivariate generalized least-squares regression (PGLS) of the stress percentile values on talar volume was performed to check that the observed results were not merely attributed to size-dependent effects.

#### 2.3.2. Average values and quasi-ideal mesh

Von Mises stress is an isotropic criterion used to predict the yielding of ductile materials determining an equivalent state of stress [34]. It has been shown that if the bone is considered as a ductile material and if isotropic material properties are used, the von Mises criterion is the most adequate for comparing stress states [35]. The von Mises stress distributions of the different tali were assessed using their average values and displayed using boxplots. New statistics that consider the non-uniformity of the mesh were calculated: (i) the mesh-weighted arithmetic mean (MWAM) and (ii) the mesh-weighted median (MWM) [36]. A more detailed description of these statistics is provided in electronic supplementary material, document S2.3. The application of boxplots for the stress and statistics derived from them (i.e. M25, M50, M75 and M95 percentiles) involves the generation of a QIM, thus allowing the display of the obtained stress values as boxplots [32].

#### 2.3.3. Analysis of the stress results

All statistical analyses were performed in R v. 3.4.0 [37]. Multivariate normality was rejected for the stress data (electronic supplementary material, document S2.4), so a non-parametric test was preferred. First, a PERMANOVA was calculated to test for differences between the groups considering all the stress percentiles together [38]. Then, pairwise PERMANOVA tests with a Holm correction for multiple comparisons were carried out to test for differences in stress values between the three locomotor categories. In both cases, Euclidean distances were used as similarity index.

### 2.4. Geometric morphometrics

Thirty Cartesian coordinates were collected on the surface of the talar models (figure 2*b*) [39,40]. These raw coordinates were analysed using the ‘geomorph’ R package [41] and are available in electronic supplementary material, file S4. A Procrustes superimposition was performed to remove the differences due to scale, translation and rotation, leaving only variables directly related to shape. Then, these shape variables were used to carry out a principal component analysis (PCA) to visualize morphological affinities. A broken-stick model was applied to determine the number of PCs to be used in the subsequent analysis. To visualize the structure of the data for both shape and stress variables, a consensus phylogeny was projected onto the space identified by the first two PCs obtained from the variance–covariance matrix of the shapes of the analysed modern taxa and the mesh-weighted median stress value (i.e. MWM) on the *z*-axis. In addition, the phylogenetic signal was estimated for both the morphometric and stress data using a mathematical generalization of the *K*-statistic appropriate for multivariate data (i.e. Kmult) [42]. A PGLS regression of talar shape on centroid size was also performed to check that the observed results were not merely attributed to allometric effects. Then, a standard PLS and a phylogenetic PLS analysis were carried out to examine the association between the shape variables and the percentile stress values [43]. PLS computes the covariation level between the two blocks of data, while the phylogenetic PLS also takes into account the phylogenetic structure of data assuming a Brownian motion model of evolution [44].

### 2.5. Fossil locomotor classification

A previous study has shown that, when using only talar shape, it was possible to distinguish between clamber/suspensory, leaper and arboreal quadruped locomotor modes [6], but it remains unexplored whether including stress information explains the differences in talar functional morphology between different locomotor modes or improves the locomotor resolution. Therefore, two different datasets were analysed and used to classify the fossil material: (i) biomechanical and (ii) morphometric data.

The biomechanical data comprised a set of 10 variables generated using the Intervals' method described in [45] (further information about this procedure can be found in electronic supplementary material, S2.5 and table S5). As a pre-processing procedure, a Box-Cox transformation was performed to normalize the interval data. In addition, these 10 intervals were centred and scaled to improve the numerical stability of some subsequent calculations and to standardize their scale. As a result of centring, the variables have a zero mean, while scaling coerce the predictors to have a common standard deviation of one. These transformed interval values were subsequently used in the classification analyses.

The morphometric data consisted of the number of PCs obtained from the broken-stick model used to assess the significance of variance. This broken-stick model showed that only the first seven PCs had eigenvalues larger than the values randomly generated by the model. These seven PCs accounted for 63.6% of the total variance of the sample, thus providing a reasonable approximation of the total amount of talar shape variation. There was no need to perform any pre-processing procedure prior to the application of the ML classification methods, given that the original raw coordinates were subjected to a Procrustes superimposition, which centred each configuration of landmarks at the origin, scaled them to unit centroid size and rotated them to optimal alignment on the average shape. In addition, a PCA was carried out using these shape coordinates to avoid any possible collinearity.

Six supervised algorithms were selected in order to represent a wide range of different classification models: (i) linear discriminant analysis (LDA); (ii) classification and regression tree (CART); (iii) *k*-nearest neighbours (KNN); (iv) naive Bayes (NB); (v) support vector machine (SVM) and (vi) random forest (RF). All the models were prepared and performed using the ‘caret’ package for R [46], which consist of a set of functions that help to streamline the generation of predictive models (https://topepo.github.io/caret/). The performance of the classification models was quantified using the confusion matrix from which the overall classification accuracy (i.e. error rate) was computed, as well as by computing Cohen's Kappa coefficient [47]. To assess the performance of the models, the complete dataset was resampled using a ‘leave-group-out’ cross-validation [48]. This method generates multiple splits of the data into modelling and prediction sets. This procedure was repeated 200 times and the data were divided into a modelling set containing 75% of randomly allocated observations, while the testing set contained the remaining 25%. The repetition number was selected to get stable estimates of performance and to reduce the uncertainty in these performance estimates. The best classification models obtained for the morphometric and biomechanical data were then used to infer the main locomotor mode of the Miocene fossil sample by computing their class probabilities to belong to each one of the locomotor categories. Further methodological details and a brief description of the classification algorithms applied here can be found in electronic supplementary material, document S2.6.

## 3. Results

### 3.1. Finite-element analysis

The PGLS of the stress percentile values on talar volume indicates that allometry is not a factor affecting our results when phylogenetic non-independence is considered (electronic supplementary material, table S2.7).

**Figure 3.**
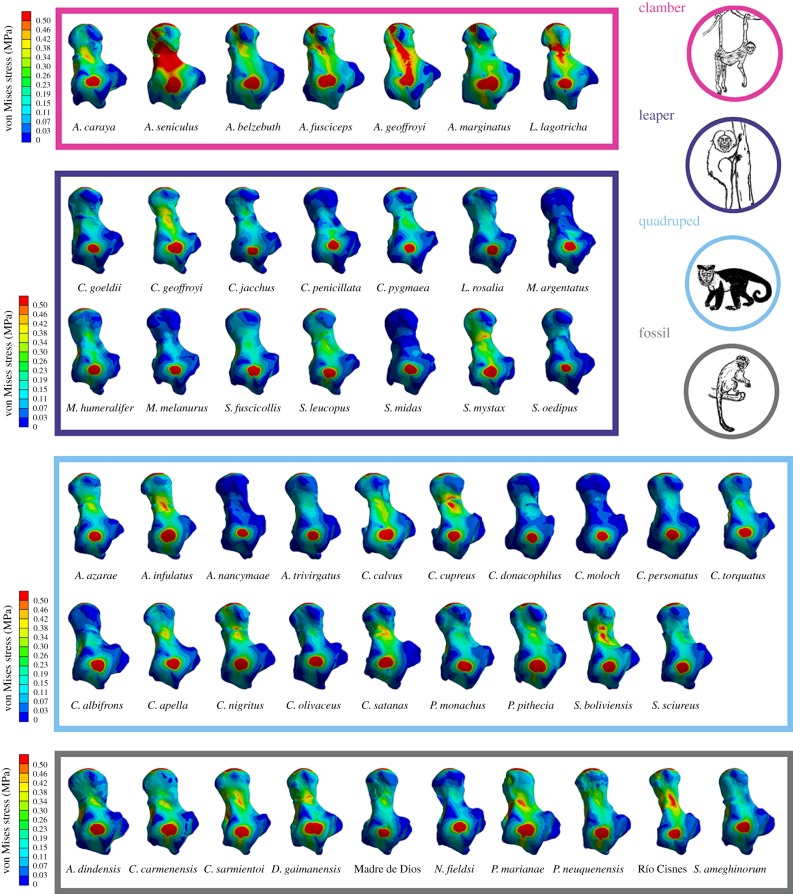
von Mises stress distribution for all the analysed specimens. (Online version in colour.)

**Figure 4.**
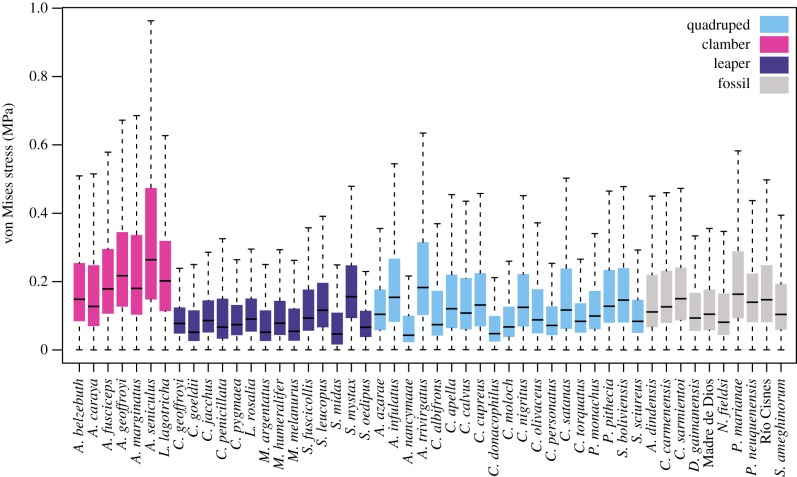
Boxplots of von Mises stress distributions for all the analysed specimens. (Online version in colour.)

Figure 3 shows stress maps for all the analysed species, while figure 4 displays the stress distribution in boxplots. The visual representation of the stress distribution for each talus is a useful indicator for comparative inference on their biomechanical behaviour, because these stress patterns can be interpreted as a sign of relative strength (i.e. specimens exhibiting higher stress levels are weaker with that defined loading pattern). The quantitative values of MWM, MWAM, the quartiles of the boxplots of stress, the PEofAM and the PEofM (i.e. percentages of error used to define the QIM) can be found in electronic supplementary material, table S3.

Figure 4 shows that when comparing locomotor behaviours in extant species, the ‘clamber/suspensory’ group exhibits the weakest tali, while the ‘arboreal quadruped’ taxa show intermediate values and ‘leaper’ species present the strongest tali. There were significant differences between groups when comparing all the stress percentiles together using the PERMANOVA (*F*: 21.437; *R*^2^: 0.54; *p*-value: 1 × 10^−4^; 9999 permutations) (table 3). Therefore, it is possible to distinguish these main locomotor behaviours using a biomechanical approach.

**Table 3.** Pairwise PERMANOVA results.

	*F*	*R*^2^	adjusted *p*-value (Holm correction)
clamber/suspensory versus arboreal quadruped	18.84	0.44	0.003
clamber/suspensory versus leaper	57.05	0.75	0.003
arboreal quadruped versus leaper	6.18	0.17	0.012

### 3.2. Geometric morphometrics

**Figure 5.**
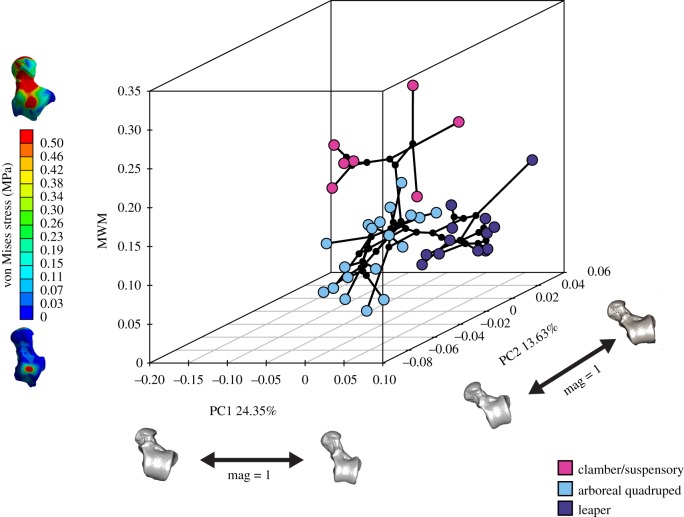
Phylomorphospace of the first two morphometric PCs and stress values (MWM) as vertical *z*-axis. One of the models closest to the mean shape was warped to match the multivariate mean using the thin-plate spline method, and then the obtained average model was warped to represent the variation along the PC axes. In addition, the von Mises stress maps of two extreme models are displayed to facilitate the understanding of the *z*-axis. (Online version in colour.)

The phylomorphospace of the first two PCs and the MWM as *z*-axis displays three main areas of occupied morphospace (figure 5), which broadly resemble the main NWM locomotor groups. PC1 mostly separates between the Atelidae on one extreme of the axis, which shows clambering/climbing and suspensory behaviours, and the Callitrichinae, displaying claw-assisted clinging postures and higher frequency of leaping behaviour towards the opposite extreme of the axis. The more specialized locomotor behaviours separated along PC1 were also separated from mainly quadrupedal species on PC2. There was a central area of more ‘generalist’ species, which are predominately quadrupedal although they engage in other locomotor behaviours, while the negative extreme of PC2 was occupied by predominantly quadrupedal species with variable, but usually moderate, rates of leaping behaviour. Finally, the MWM *z*-axis mostly separated between the clamber/climbing Atelidae (which shows higher stress values) from the rest of the species. A two-dimensional plot of the phylomorphospace is also provided to facilitate the visual inspection of the morphometric results (electronic supplementary material, figure S6).

**Figure 6.**
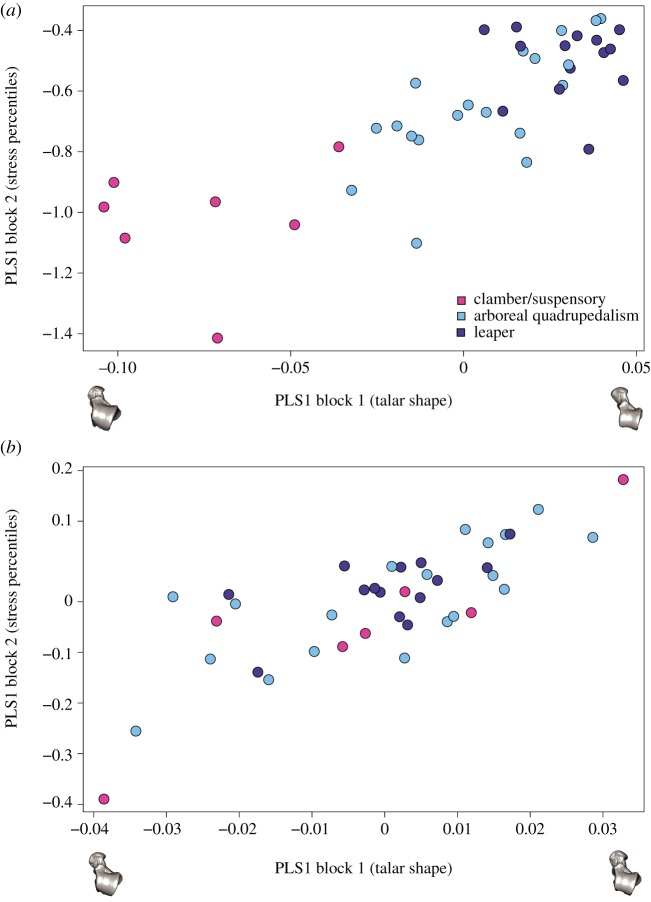
(*a*) Standard PLS and (*b*) the phylogenetic PLS analysis of the shape variables and stress percentile values. One of the models closest to the mean shape was warped to match the multivariate mean using the thin-plate spline method and then the obtained average model was warped to represent the covariation between the two blocks of data for PLS1. (Online version in colour.)

Significant phylogenetic signal was found for both morphometric (Kmult: 0.34972; *p*-value: 1 × 10^−4^; 9999 permutations) and biomechanical data (Kmult: 0.32716; *p*-value: 0.0158; 9999 permutations). We found an extremely weak and not significant association between talar shape and centroid size when taking into phylogenetic information (electronic supplementary material, S2.8); hence, talar shape variation cannot be merely attributed to evolutionary allometric effects. The percentile stress values (i.e. M25, M50, M75 and M95) showed significant covariation with talar shape (r-PLS: 0.8; *p*-value 2 × 10^−4^; 9999 permutations), as well as when considering the phylogenetic information (phylogenetic r-PLS: 0.78; *p*-value: 0.0018; 9999 permutations) (figure 6*a* and *b*, respectively). This means that there is a strong association between talar shape and the biomechanical performance of the talus.

### 3.3. Fossil locomotor classification

**Figure 7.**
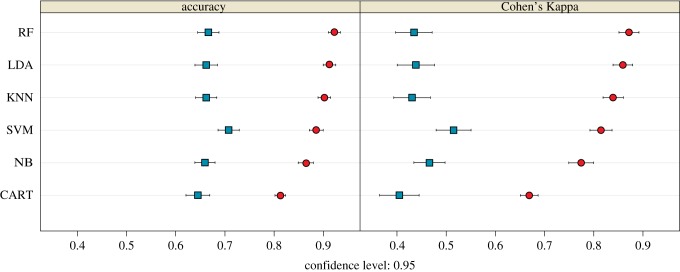
Dot-plot comparing the accuracy and Cohen’s Kappa values of the different classification models applied to biomechanical (blue squares) and morphometric (red dots) data. The dots represent the average accuracy and Cohen’s Kappa values after performing the ‘leave-group-out’ cross-validation (200 repeats), while the whiskers display their respective 0.95 confidence level. Model acronyms: RF, random forest; LDA, linear discriminant analysis; KNN, k-nearest neighbours; SVM, support vector machine; NB, Naive Bayes; CART, classification and regression trees. (Online version in colour.)

**Figure 8.**
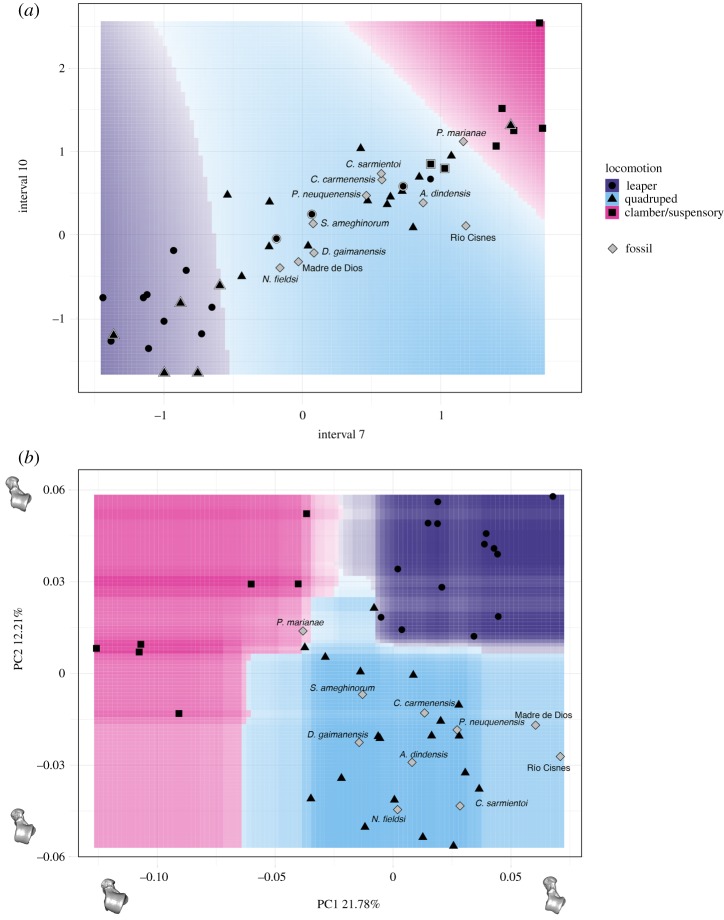
Decision boundary plots for (*a*) biomechanical and (*b*) morphometric data. In (*a*), only the seventh and 10th intervals are displayed because they contribute the most to class separation, while in (*b*) only the first two PCs are shown. The space is coloured depending on what locomotor category the (*a*) SVM or the (*b*) RF algorithm predict that region belongs to, whereas the lines between coloured areas represent the decision boundaries. Colour intensity indicates the certainty of the prediction in a particular graph area (i.e. darker colours imply a higher probability of belonging to a particular class). Symbols surrounded by a white rim represent misclassified specimens. In (*b*), one of the models closest to the mean shape was warped to match the multivariate mean using the thin-plate spline method, and then the obtained average model was warped to represent the variation along the two PC axes. (Online version in colour).

Figure 7 shows the accuracy and Cohen's Kappa results for all the tested models for both the biomechanical and morphometric data after performing the ‘leave-group-out’ cross-validation and using the automatic grid search. Shape data outperformed interval stress data when classifying according to locomotion in both accuracy and Cohen's Kappa values. The most accurate model for the biomechanical data was the SVM using a linear kernel, while in the case for the morphometric data, the most accurate model was the RF. The only tuning parameter in the biomechanical SVM model using a linear kernel is ‘cost’, so we expanded the grid search to consider more values; however, the best result was still achieved when cost = 2 (average accuracy: 0.708; average Cohen's Kappa: 0.515) (figure 8*a*). A Cohen's Kappa value of approximately 0.5 represents a reasonable agreement [47]; therefore, we used the best obtained model to classify the fossil sample (SVM model using biomechanical data as described in table 4). Using these interval data, all the fossil specimens were classified as arboreal quadrupeds. However, it is important to note that *Paralouatta marianae* showed quite similar values for both the arboreal quadruped and clamber/suspensory categories (SVM model using biomechanical data as given in table 4). In addition, although *Cebupithecia sarmientoi* and *Proteropithecia neuquenensis* were classified as arboreal quadrupeds, they also showed important posterior probabilities for the leaper category.

**Table 4.** Prediction results for the fossil sample.

species/specimen	SVM model using biomechanical data			RF model using morphometric data		
	posterior probabilities			posterior probabilities		
	leaper	arboreal quadruped	clamber/suspensory	leaper	arboreal quadruped	clamber/suspensory
*Aotus dindensis*	0.07	0.71	0.22	0.03	0.92	0.04
*Carlocebus carmenensis*	0.15	0.68	0.17	0.05	0.93	0.02
*Cebupithecia sarmientoi*	0.37	0.46	0.18	0.04	0.89	0.07
*Dolichocebus gaimanensis*	0.13	0.79	0.08	0.02	0.97	0.01
Madre de Dios	0.32	0.59	0.09	0.15	0.74	0.11
*Neosaimiri fieldsi*	0.24	0.68	0.08	0.01	0.98	0.01
*Paralouatta marianae*	0.09	0.46	0.45	0.21	0.36	0.42
*Proteropithecia neuquenensis*	0.41	0.43	0.17	0.05	0.94	0.01
Río Cisnes	0.13	0.62	0.25	0.08	0.79	0.13
*Soriacebus ameghinorum*	0.22	0.68	0.10	0.01	0.99	0.00

The obtained RF model for the morphometric data was further tuned using a manual grid search. Two parameters were tuned in this model, the number of tress to grow (i.e. 100, 200, 500, 1000 and 2000) as well as the number of variables randomly sampled as candidates at each split (i.e. 2, 3, 4, 5 and 6). In general, the RF model was quite robust when changing these tuning parameters, showing similar classification accuracies. The final best RF model grew 200 trees and used five variables randomly sampled as candidates at each split (average accuracy: 0.925; average Cohen's Kappa: 0.876) (figure 8*b*). By applying the final RF model, the fossil sample was classified (RF model using morphometric data as presented in table 4), and all the specimens were categorized as arboreal quadrupeds excepting *Pa. marianae*, which was classified as a clamber/suspensory individual. Briefly, discussed results for each one of the analysed fossils can be found in electronic supplementary material, document S2.9.

## 4. Discussion

Studying the functional morphology of the platyrrhine talus is important because it represents one of the few post-cranial structures available in many of the oldest platyrrhine fossils, but also because its morphology has been shown to reflect locomotor behaviour [6] and is associated with biomechanical performance (figure 6*a*,*b*). The biomechanical data obtained from the FEA modelling show that the ‘clamber/suspensory’ species exhibit significantly higher stresses than the other two analysed locomotor categories, while the ‘leapers’ show the lowest stress values. This could be explained by the fact that leaping would be expected to exert higher forces on the lower extremities because the accelerations in primate leaping are generally high (for a review, see [36]). By contrast, suspensory behaviours would exert comparatively reduced bending forces on the limb bones [49], which is relevant when considering that bending has been shown to be the loading pattern that most commonly leads to high stresses in limb bones [50]. In addition, it has also been shown that repetitive loading can cause bones to fail at much lower loads [51,52]. To avoid the possible damage caused by the effect of fatigue, it is plausible that talar morphologies that reduce stress would have been selected for in leapers [7,9,10]. A recent study has shown that platyrrhine talar morphology seemed to evolve towards three different selective optima [6], which are related to the main ecophylectic groups observed in extant NWM.

The morphometric analysis clearly distinguished in PC1 between the species showing frequent leaping from those with adaptions for clamber/suspensory behaviour, while PC2 distinguished the most quadrupedal species from the rest. The talar morphology of the species exhibiting leaping can be described as showing an anteroposteriorly shorter trochlea with more parallel medial and lateral rims and a longer anterior calcaneal facet. This morphology was the strongest one in the biomechanical analysis (figure 3). On the other hand, the weakest talar morphology, which is associated with clamber/suspensory behaviours, included characters such as a broader head, greater trochlear wedging, a lower trochlea and a shorter anterior and longer posterior calcaneal facet. The lower stress values observed in leapers can be explained due to their mediolaterally broader trochlea with lateral and medial rims and robust talar body, which better distributes the applied load on the trochlear surface. By contrast, the clamber/suspensory group shows a morphology characterized by a more ‘wedged’ trochlea with a low trochlear relief, which maximizes the mobility at the talocrural joint, but at the cost of increasing the stress on the trochlear surface.

The PLS analyses showed that there is an association between talar shape and stress values. A previous study has shown that there is also a significant association between locomotor data and talar morphology [6]; therefore, the present results contribute to the understanding of the relationship between talar morphology and locomotor behaviour by providing the link between these two factors: the biomechanical behaviour of talus during locomotion. The talus acts as the main mechanical link between the leg and the foot [30], transmitting not only the forces derived from an animal's body mass but also providing stability and mobility for the posterior limbs during diverse postural and locomotor behaviours [7]. These behaviours probably exert differential loading regimes on the talus, thus gradually shaping its morphology. It is well known that the talus is primarily stiffened by trabecular networks that are remodelled influenced by mechanical loading [30], and that trabecular architecture can be informative about locomotor differences among different taxa [53–55]. Although we were limited by our fossil sample, future studies could include trabecular information as part of the simulated loading scenarios to further explore the link between ecomorphology and biomechanics.

When comparing the two techniques (i.e. FEA and GM) in the classification task, using several ML algorithms, the best performing approach was an RF model applied to GM data. Even though we were concerned with functional groupings, we found that shape outperforms FEA-derived values when classifying according to locomotor groups. This is likely because morphological variation is influenced by diverse factors, including loading, diet, sex and evolutionary history, among others, all of which may be associated with differences in locomotion. A complex phenomenon such as the differences in locomotor behaviour reflected in talar morphology probably includes many factors that are only partially accounted for when biomechanical analyses are performed. These kinds of analyses simply focus on more specific and constrained aspects of variation (e.g. loading resistance), whereas GM incorporates more diverse sources, although with the disadvantage of not always knowing what part of this variation is strictly related to function. The main value of biomechanical approaches is that they enable us to test ideas about the adaptive value of particular features of the fossils, in ways that associative statistical analysis alone cannot. This is when mechanical analyses such as FEA are required to test alternative functional hypotheses, making both approaches complementary. However, it is important to bear in mind that the load cases chosen only allow the FEA to consider specific aspects of function (e.g. stresses arising from specific loadings) and so may omit important functional differences that would require different measures of load resistance or different simulated load cases to characterize them. Therefore, it is possible that the functional analysis performed here failed to identify some functionally relevant differences between groups. A more detailed biomechanical scenario might yield better discriminating results when comparing locomotor groups, so future studies should test other loading scenarios that might improve discriminatory performance, including the possibility of generating load cases using multi-body dynamic analysis [56].

It is important to keep in mind that when reconstructing locomotor behaviours in fossil taxa, it is the main locomotor modes that are reconstructed and not the entire repertoire of possible habits [57]. Both the biomechanical and morphometric-based classifications categorized most of the fossil sample as arboreal quadrupeds, which is consistent with previous proposals based on morphological analyses, morphometric classifications and ancestral state reconstructions [6]. It is interesting that in spite of the class imbalance that could affect our results, *Paralouatta* is classified as a possible clamber/suspensory species using the morphometric data. However, this taxon also showed not negligible posterior probabilities for the other two tested locomotor modes, thus probably indicating a mixed locomotor pattern. Previous analyses have shown that its talar morphology shows some similarities with the Alouattinae (which are species that spend an important amount of time exhibiting clamber/suspensory behaviours) and some of the oldest Patagonian fossils (i.e. *Dolichocebus*, *Carlocebus*, *Soriacebus*; which are specimens reconstructed as mostly quadrupedal) [6]. Based on the presence of a strong cotylar fossa, along with several other post-cranial adaptations, it has been suggested that *Paralouatta* could even have been a semi-terrestrial species [57]. The present analysis did not include this category so it was not possible to rule out this possible locomotor specialization, but the fact that our analysis indicates different locomotor modes probably points to locomotor behaviours similar to *Alouatta* (i.e. showing variable degrees of arboreal quadrupedalism, climbing and clambering)*.* It is also interesting that even though the Madre de Dios talus was classified as a quadruped, its posterior probabilities suggest a variable degree of leaping behaviours as has been previously proposed [6]. In addition, the biomechanical results suggest that *Proteropithecia* could have engaged in a significant amount of leaping, which is consistent with previous suggestions [58]. A limitation of the present analyses is that they rely on extant platyrrhines to assess the postural behaviour of some species that might be located outside this monophyletic group (e.g. stem taxa or long isolated primitives like the Caribbean forms) and that could have exhibited unique locomotor adaptations not represented by the locomotor categories analysed here. Nevertheless, we analysed important primate postural behaviours that can contribute to future fossil locomotor interpretations.

We were able to classify the fossil sample into broad locomotor categories, providing information regarding some aspects of the positional behaviour of Miocene platyrrhines. However, until finding post-cranial remains for the platyrrhine fossils from the Eocene and Oligocene, not much can be inferred with certainty about the ancestral locomotor condition of the first NWMs. Although the present analyses cannot provide definitive answers about the ancestral locomotor condition of platyrrhines, they do provide relevant information about the following step in the evolutionary history of NWMs. The present results indicate that most fossil specimens exhibit a generalist and possibly primitive morphology, while showing significant size variation (e.g. Madre de Dios: 352 g; *Pa. marianae*: 4708 g), and the biomechanical and morphometric data are consistent in classifying most fossil individuals as arboreal quadrupeds. Previous analyses have shown that after an initial diversification in size, platyrrhine talar shape seemed to gradually evolve towards three different selective optima, represented by the three main locomotion habits observed in extant NWM [6]. Therefore, this could imply that the Miocene sample could be representing an ancestral quadrupedal condition prior to the subsequent locomotor diversification observed in platyrrhines [6].

Ecomorphological studies have provided numerous morphological correlates of ecological, functional and/or locomotor categories (e.g. [6,59–61]). Some of these morphological traits allow discrimination based on these kinds of categories, enabling us to make inferences about possible adaptations in extinct taxa. Nonetheless, absolute discrimination among such categories is rarely achieved by any single measurement or set of variables because these values normally show considerable overlap. This overlap is a direct consequence of the covariation pattern observed in most morphological adaptations. This means that in many cases, the way in which any morphological feature adapts might be also influenced by the changes occurring in other regions of an animal's morphology and by other environmental factors besides the one under analysis. The implication of this widespread covariation is that many ecomorphological adaptations might be better characterized by complex morphological patterns that can be better described in a multi-dimensional morphospace rather than defined by single variables or indices. These multi-dimensional spaces cannot be simply displayed in two dimensions, so traditionally multivariate techniques such as PCAs or LDAs have been commonly applied to deal with this sort of classification problems. However, more recently, ML approaches have been used to tackle these sorts of problems due to their inherent capabilities when it comes to uncover patterns, associations and statistically significant structures in high-dimensional data [14]. This study showed how using different ML algorithms is possible to successfully address problems of group analysis and classifications using morphometric and biomechanical data. The present findings have shown that the application of these algorithms to at least some types of morphometric and biomechanical problems is a contribution that can improve the traditional way classification tasks have been undertaken in these fields. Some of the advantages are evident, such as the flexibility that allows the use of several different algorithms which can have dissimilar performance depending on the specific problem, rather than using only one classification approach (e.g. LDA) without comparing its performance against alternative approaches that might be more suitable for a particular task. The choice of an algorithm is an active area of research within the ML field and depends on the characteristics of the data-space being searched. Incorporating the predictive modelling techniques derived from ML into the standard virtual functional morphology toolkit can prove to be a useful addition that could offer further flexibility and predictive power when analysing data and dealing with classification and regression problems.

## Supplementary Material

Phylogeny

## Supplementary Material

Alignment procedure prior to FEA; Case-specific fossil reconstruction procedures; Description of the applied FEA statistics; Normality test for the stress percentiles; Further details about the Intervals' method; Further methodological details and brief description of the machine learning algorithms applied to classify the fossil sample into broad locomotor categories; a) PGLS of stress on talar volume; PGLS of talar shape on centroid size; Fossil-specific results

## Supplementary Material

Finite element model information and stress results

## Supplementary Material

Talar 3D coordinates

## Supplementary Material

Convergence procedure applied as part of the Intervals’ method

## Supplementary Material

Phylomorphospace of the extant platyrrhine sample computed using the morphometric data.

## Data accessibility

Further explanations about certain procedures, as well as additional numerical results supporting this article, have been uploaded as part of the electronic supplementary material. The phylogeny and 3D raw coordinates are also available. The R scripts used to carry out the different analyses are available upon request.
