# Supplementary material for: Inferring locomotor behaviours in Miocene New World monkeys using finite element analysis, geometric morphometrics and machine-learning classification techniques applied to talar morphology
Source: J R Soc Interface. 2018 Sep 26;15(146):20180520. doi: 10.1098/rsif.2018.0520 (PMC6170775; doi:10.1098/rsif.2018.0520)
Supplement: Alignment procedure prior to FEA; Case-specific fossil reconstruction procedures; Description of the applied FEA statistics; Normality test for the stress percentiles; Further details about the Intervals' method; Further methodological details and brief description of the machine learning algorithms [file rsif20180520supp2.pdf]

Document S2. Further details about several methodological procedures

S2.1 Alignment procedure

The tali were aligned according to the standard anatomical position to allow meaningful comparisons between specimens, since it has been shown that the fabric orientation in the talar body indicates that, practically, all primate taxa show a generally consistent pattern of orientation restricted primarily to a dorsoplantar direction [1]. In order to avoid possible problems when aligning different individuals according to the anatomical standard plane (due to inter-specific morphological differences), we selected one individual as a reference (i.e., *Chiropotes satanas*) to perform a best-fit alignment using Geomagic Studio® (3D Systems, v. 12, Rock Hill, SC, USA) in order to align all the models with respect to the common reference plane described above. This procedure was carried out prior to FEA to align all the models so that loads could be applied in the same axis and to allow an easier interpretation of the stress results. The procedure involved fitting two talar models at each time by measuring from point to point and adjusting the location of the target model to the stationary reference specimen until the average deviation was as low as possible using an iterative procedure (iteration number: 9,999). The sums of squares of the distances between the sample pairs were minimized over all the rigid motions that could realign the two models to attain the best-fit alignment between them. This process was repeated for each one of the analysed specimens to ensure that all the models were standardise in the same orientation, thus allowing comparisons.

S2.2 Case-specific fossil reconstruction procedures

Madre de Dios: This specimen exhibited some minor cracks on its surface and a relatively small hole on its trochlear surface. Hence, it was manually repaired using the 'sculpt knife' in Geomagic Studio® in order to correct these minor defects (sculpt knife parameters: width: 0.1 mm; offset: 0.01 mm; smoothness: 30; shape: 0).

Dolichocebus gainamensis: This fossil talus showed an eroded talar head, along a noticeable missing portion of the trochlear surface. Manually reconstructing these missing portions would have been particularly subjective, due to the size of the damaged areas; hence it was decided to avoid that approach. Therefore a reference-based geometric reconstruction tactic was preferred instead [2–4]. The consensus shape of the comparative extant sample was estimated and then the 3D surface of a *Chiropotes satanas* specimen was warped to match this multivariate mean configuration using the thin plate spline interpolation function. This specimen was selected because a previous analysis of 203 platyrrhine tali [5] has shown that this species exhibits one of the closest morphologies to the talar consensus configuration, and also due to its high resolution that allowed us to carry out the different proposed reconstruction approaches. This consensus surface model was then warped to match the coordinates of *Dolichocebus gainamensis*. In this way, missing data was estimated by mapping this consensus configuration to the fossil specimen with missing landmarks [3] by applying again the thin plate spline interpolation function based on the subset of observable landmarks (the landmarks 3, 4 and 25-29 were not used because they were absent in the fossil individual). These warping procedures were carried out in Landmark v. 3.6 [6]. Subsequently, the obtained

model was imported into Geomagic Studio® and scaled to the same volume as *Dolichocebus gainamensis*. Then it was aligned with respect to it, using a best-fit alignment (sample size: 10,000; tolerance: 0.17699). Then the damaged portions were removed from the fossil and the reconstructed areas were trimmed from the warped model and used to patch the removed areas. Finally, the patched areas were slightly smoothed by using the 'sand paper' tool in the same software.

Soriacebus ameghinorum: This specimen was entirely missing the posterior calcaneal articular surface. The same procedure outlined above for the *Dolichocebus gainamensis* specimen was performed in order to reconstruct the missing anatomical portion using the *Chiropotes* surface file already described. The subset of landmarks used to perform the thin plate spline warpings considered all the original coordinates except for landmarks 19-24. Additionally, using the warped model some areas of the talar neck and medial tubercle were reconstructed.

Río Cisnes: This fossil talus did not exhibit any extremely broken areas, however its whole surface was noticeably eroded. For that reason it was not possible to apply any of the previously described reconstruction approaches. In this case, it was decided that instead of manually and subjectively reconstructing the eroded surfaces, it was better to simply use the already described consensus surface model and warp it to match the available landmarks for this specimen.

S2.3 Description of the Mesh-weighted arithmetic mean (MWAM) and Mesh-weighted median (MWM) statistics

Following the proposal stated in [7], it is possible to defined the Arithmetic Mean (AM) in the context of stress results obtained from FEA, as be the sum of the value of the von Mises stress (σ_{VM}) of each element divided by the number of elements of the mesh (Equation 1).

Equation 1.

$$AM = \frac{\sum_{i=0}^{n} \sigma_{vM}}{n}$$

Mesh-weighted arithmetic mean of stress distribution (MWAM): This statistic corresponds to the sum of the value of the von Mises stress (σ_{vM}) for each element multiplied by its own volume (V) and divided by the total volume (Equation 2). Some data points contribute more than others depending on the size of the element in the MWAM.

Equation 2.

$$MWAM = \frac{\sum_{i=0}^{n} ({\sigma_{vM}}^{i} \cdot \mathrm{V}^{i})}{\sum_{i=0}^{n} \mathrm{V}^{i}} = \frac{\frac{\sum_{i=0}^{n} ({\sigma_{vM}}^{i} \cdot \mathrm{V}^{i})}{n}}{\frac{\sum_{i=0}^{n} \mathrm{V}^{i}}{n}} = \frac{AM({\sigma_{vM}}^{i} \cdot \mathrm{V}^{i})}{AM(\mathrm{V}^{i})}$$

Equation 2 shows that MWAM is equivalent to the division of the arithmetic mean of the product of stress and volume by the arithmetic mean of the volume, which is easier to calculate and does not need the correction of the weight element by element. If all weights are equal, then the weighted mean exactly

corresponds to the arithmetic mean. Consequently, AM and MWAM will be the same in a uniform mesh.

Mesh-weighted median of stress distribution (MWM): The median corresponds to the value of the data that has an equal number of items on either side of it (it divides a frequency distribution into two halves), after arranging the data in order of magnitude (i.e., ranks). In the context of FEA, the median would represent the value that divides the higher half from the lower half of the values of von Mises stress values distribution after they have been ordered. Based on the formulation presented in Equation 2., the Mesh-Weighted Median (MWM) of a stress distribution can be defined as the division of the median of the product of stress and volume by the median of the volume (Equation 3).

Equation 3.

$$MWM = \frac{median({\sigma_{vM}}^{i} \cdot \mathrm{V}^{i})}{median(\mathrm{V}^{i})}$$

Percentage error of the arithmetic mean (PEofAM) and percentage error of the median (PEofM): MWAM and MWM are also required to compute the percentage error of the arithmetic mean (PEofAM) and percentage error of the median (PEofM), which are values needed to ensure that the models were good-enough QIMs as described in [7]. These two error statistics evaluate the

difference between the non-weighted value and the weighted value of the mean and the median (PEofAM in Equation 4 and PEofM in Equation 5), respectively.

Equation 4.

$$PEofAM = \left(\frac{MWAM - AM}{MWAM}\right) \cdot 100$$

Equation 5.

$$PEofM = \left(\frac{MWM - M}{MWM}\right) \cdot 100$$

By ensuring a QIM, we are able to use the stress values in the percentiles 25%, 50%, 75% and 95% of the von Mises stress distribution as talar strength proxies as well. The highest value of the boxplot was not considered since unusually high stresses appear where the boundary conditions are located. These stresses are artificially elevated, tending to infinity, by the constraints exerted on the model due to a numerical singularity [8]. This numerical singularity results from the mathematical approach applied, and not to any meaningful biological process. Consequently the highest results from these areas were not considered, and instead we used the 95% percentile as a peak stress [9].

S2.4 Normality test for the stress percentiles

A Mardia's multivariate normality test was applied to check the assumption of multivariate normality in the distribution of the stress percentiles (i.e., M25,

M50, M75 and M95). It was found that the stress percentile data was not multivariate normal (g1p: 5.48, skewness Chi-square: 36.53, p-value: 0.013; g2p: 28.61, kurtosis Z: 2.11, p-value: 0.035), therefore non-parametric statistics (e.g., PERMANOVA) were preferred to analyse the stress data.

S2.5 Further details about the Intervals' method

This recently published methodology divides the values of stress of all the elements of the model into different *N* intervals, each one of them corresponding to the amount of volume of the original model having a specific range of stress values [10]. These percentages are computed in relation with the total volume of the model of each specimen for standardisation. The number of intervals to be analysed (i.e., the number of biomechanical variables) was chosen following the convergence procedure proposed by [10], in which several PCAs are performed using a different number of intervals (in this case: 5, 10, 25, 50, 75 and 100) in order to establish the threshold in which the data converged (i.e., when adding more intervals yielded similar patterns in the PCAs and when the correlation between the respective first PCs was higher than 0.99). The obtained results from this procedure showed that convergence was quickly obtained by using just 10 intervals (Table S5).

S2.6 Further methodological details and brief description of the machine-learning algorithms applied to classify the fossil sample into broad locomotor categories.

A technical outline of the different applied algorithms is far beyond the scope of this study and several introductory books cover the topic (e.g., [11,12], along with the 'caret' package webpage https://topepo.github.io/caret/. Nonetheless, we have provided below further technical details about the applied ML approach, as well as brief description of the ML algorithms that were used.

Additional information about the applied machine-learning procedures

Parameter tuning:

The 'caret' package also provides a grid search (automatic and manual) where it is possible to specify tuning parameters for the models. We first started with an automatic grid search by setting the 'tuneLength' option to indicate the number of different values to try for each algorithm parameter (we initially set this parameter to 10 in all the tested models). This only supports integer algorithm parameters, thus providing a quick first guess as to what values to try and which models are more promising. Then the models that were the most accurate for each one of the datasets (i.e., biomechanical and morphometric data) were further tuned by setting a manual grid search. In the grid, each algorithm parameter was specified as a vector of possible values. These vectors were combined to define all the possible combinations to try in order to further improve the performance of the model. Then using the best final model the fossil sample was classified into the different locomotor categories by computing the class probabilities of belonging to each one of the categories.

Cohen's Kappa:

In addition, it was also mentioned that Cohen's Kappa was calculated as a performance measurement. This statistic can range between −1 and 1, where a value of 0 means that there is no concordance between the observed and predicted classes, whilst a value of 1 would indicate perfect agreement of the model prediction and the observed classes (negative values are indicative that the prediction is in the opposite direction of the truth, but large negative values are rare when dealing with predictive models).

Brief description of the applied algorithms

Numerous models have been developed not only to analyse continuous variables but purposely focused or adapted to deal with categorical responses [13]. In machine-learning, classification refers to the task of identifying to which of a set of classes/categories a new observation (e.g., specimen) belongs, based on a training dataset comprising observations whose class/category membership is already known [14]. Classification models typically generate two kinds of predictions. In a similar fashion as regression models, classification models compute a continuous valued prediction that often takes the form of a probability or probability-like value (i.e., the predicted values of class/category membership for any individual sample/observation range between 0 and 1 and sum to 1) [15]. Additionally, classification models also produce a predicted class in the form of a discrete category, which helps to allocate observations. Below a brief description of the models that were applied in the present work is provided, but interested readers are encouraged to search for primary literature on the topic for more detailed technical aspects and further information.

-Linear discriminant analysis (LDA): There are several methods available within the discriminant analysis family, but the best known corresponds to the LDA, developed by [16] and [17] in different but equivalent approaches. Based on [16], the idea is to find the linear combination of the variables such that the between-group variance is maximised relative to the within-group variance. This means that the objective is to find the combination of the variables that provides the maximum separation between the centres of the data, whilst at the same time minimising the variation within each group of data [16,17]. Mathematically, let B represent the between-group covariance matrix, whereas W correspond to the within-group covariance matrix. Then the abovementioned problem can be expressed as finding the value of *b* such that

Equation 6.

$$\frac{b'Bb}{b'Wb}$$

is maximised. The solution to this optimization problem is the eigenvector corresponding to the largest eigenvalue of $W^{-1}B$. This vector is a linear discriminant, and following discriminants are found via the same optimization procedure subject to the constraint that the new directions must be uncorrelated with the previous discriminants.

-K-nearest neighbour (KNN): KNN uses a sample's 'neighbourhood' to classify observations [18]. When KNN is applied for classification, it predicts the class of an observation by using the K-closest samples from the training set, where K

refers to the number of neighbours that were used to determine class membership [19]. The value of K can be arbitrarily defined by the user or tuned by applying different approaches (e.g., using a grid search). “Closeness” between observations is established by computing a distance metric (e.g., Euclidean, Minkowski, Manhattan, Canberra, among many others), whilst the choice of this metric depends on the characteristics of the variables [18]. Irrespective of the computed distance metric, it is essential to recall that the original scales of the variables affect the resulting distance calculations. Therefore, if the variables are on different scales, the computed distance metrics between observations will be biased towards variables with larger scales. Hence, to allow each predictor to contribute equally to the distance calculation, it is recommend to centre and scale all variables prior to carrying out KNN [11].

-Support vector machine (SVM): Support vector machines are a family of statistical models that have evolved considerably into one of the most versatile machine learning algorithms available [11,20], and [21] provides a comprehensive treatment. In fact, SVMs are considered one of the most successfully applied techniques for pattern recognition [22]. SVMs are based on machine learning theory, which is traditionally concerned with balancing empirical risk and the capacity of a learning machine in order to obtain a small actual risk (i.e., a good generalization performance) [21]. In brief, SVM tries to define what is the best decision boundary to separate classes given the existence of many possible decision boundaries that are capable of separating all the training samples into classes correctly [10]. Among all these possible decision boundaries, SVMs find the one that achieves maximum margin between the

categories. The margin is defined as the distance between a planar decision surface that separates the classes and the closest training samples to the decision surface. SVMs are part of a wider group of techniques known as kernel methods that owe their name to the use of kernel functions [12]. These functions enable these algorithms to work in a high-dimensional space without estimating the coordinates of the data in that space, but rather by just computing the inner products between points in a suitable feature space, which reduces computational cost even in very high-dimensional spaces [23]. Different SVM algorithms use different types of kernel functions, such as linear, radial, polynomial, among other possible kernels [24]. The kernel trick allows the SVM model produce extremely flexible decision boundaries. The choice of the kernel function parameters and the cost value control the complexity and should be tuned appropriately so that the model does not over-fit the training data.

-Naïve Bayes (NB): NB is a relatively simple technique for generating classification models that allocate observations according to class/category membership to problem instances, represented as vectors of feature values, where class membership is drawn from some finite set [11]. NB is not just one unique algorithm but also a family of related algorithms based on certain common principles [25]. For example, all NB models assume that the value of a particular variable is independent of the value of any other variables, given the class/category membership [11]. NB is based on Baye's rule, which basically computes the probability that the outcome belongs to a certain class membership, given a set of variables [26]. More mathematically, given a problem instance to be classified, represented by a vector $\mathrm{x} = (x_1, \dots, x_n)$, representing

some n features (i.e., independent variables), Baye's rule will assign to this instance probabilities

Equation 7.

$$p(C_K|x_1, \dots, x_n)$$

for each of K possible outcomes or classes C_K. Using Bayes' theorem, the above conditional probability can be written as

Equation 8.

$$p(C_K|\mathrm{x}) = \frac{p(C_K)p(\mathrm{x}|C_K)}{p(\mathrm{x})}$$

In other words, given the vector of variables x, what is the probability that the outcome is the Kth class? (i.e., the goal is to estimate $p(C_K|\mathrm{x})$). Class probabilities are created and the predicted class/category is the one related to the largest class probability [27]. The core of the model is the estimation of the conditional and unconditional probabilities associated with the set of variables. For continuous predictors, it is possible to select simple distributional assumptions, such as normality [11]. However, more complex non-parametric densities can be applied to generate more flexible probability estimates. Despite their naive design and simplified assumptions, NB classifiers have performed surprisingly well in many complex real-world situations [28].

-Classification and regression trees (CART): CART models are achieved by recursively partitioning the data space and fitting a simple prediction model within each partition [29]. As result of this recursive process, the partitioning can be graphically displayed as a decision tree [30]. Classification trees are intended for dependent variables that take a finite number of unordered values (i.e., categorical categories), with prediction error measured as misclassification cost. On the other hand, regression trees are designed for dependent variables that take continuous or ordered discrete values, with prediction error typically measured by the squared difference between the observed and predicted values [29]. The aim of CART is to partition the data into smaller, more homogeneous groups [30]. In the context of decision tress, homogeneity means that the nodes of the split are more pure (i.e., contain a larger proportion of one class in each node). A simple way to define purity in classification is by maximizing accuracy or equivalently by minimizing misclassification error. However, due to certain limitations other measures of purity are also applied. C4.5 and CART are two classification tree algorithms that follow this approach [29]. C4.5 uses entropy for its impurity function, whereas CART uses a generalization of the binomial variance called the Gini index [11].

-Random forest (RF): RF or random decision forests are an ensemble learning method for classification and regression that work by generating several decision trees during training, to then output the class that is the mode of the classes (i.e., classification) or mean prediction (i.e., regression) of the individual trees [11]. In simpler words, RF is a machine-learning method that generates multiple decision trees and merges them together to get a more accurate, robust

and stable prediction. RF are a combination of tree predictors/variables such that each tree depends on the values of a random vector sampled independently and with the same distribution for all trees in the forest [31]. Each one of the trees in the forest 'votes' when classifying of a new observation/sample, and the proportion of votes in each category across the ensemble is the predicted probability vector. Whereas the kind of tree changes in the algorithm, the tuning parameter of number of randomly selected variables to choose from at each split is the same [32]. As in regression, the idea behind randomly sampling predictors during training is to decorrelate the trees in the forest [11].

Table S2.7 Results of the phylogenetic multiple regression (PGLS) of von Mises stress percentile values on volume (9,999 iterations) carried out to test that the observed differences in von Mises stress results between the different platyrrhine taxa were not merely attributed to size-dependent effects.

	Df	SS	MS	R^2	F	Z	Pr(>F)
Volume	1	0.023	0.023	0.043	1.703	1.129	0.116
Residuals	38	0.518	0.014				
Total	39	0.541					

Table S2.8 Results of the phylogenetic multiple regression (PGLS) of talar shape on centroid size (9,999 iterations) carried out to test that the observed differences in talar shape between the different platyrrhine taxa were not merely attributed to size-dependent effects.

	Df	SS	MS	R^2	F	Z	Pr(>F)
Centroid size	1	0.002	0.002	0.020	0.777	1.407	0.086
Residuals	38	0.105	0.003				
Total	39	0.107					

S2.9 Results of the fossil sample

Aotus dindensis: The analysed specimen of *A. dindensis* was also found in the site of La Venta, Colombia [33]. Due to its particular morphological characteristics, it as was defined as member of the genus *Aotus*, although *A. dindensis* is smaller and slightly more square-shaped [34]. The morphological features of this talus have been usually inferred as indicative of arboreal quadrupedalism [35], with no evident traits that could be interpreted as signs of frequent climbing or leaping [34]. The classification results obtained by us indicate that *A. dindensis* was an arboreal quadruped (Table 4), whereas its stress results are similar to *C. apella, C. nigritus, C. calvus* and *C. cupreus* (Fig. 4). *Aotus dindensis* is located in the morphometric PCA near *C. albifrons S. boliviensis* and *C. torquatus*, which are also primarily quadrupedal species (Fig. 8b).

Carlocebus carmenensis: *C. carmenensis* represents a NWM from Pinturas, which is noticeably larger than *Soriacebus* [36]. Fortunately, four well-preserved tali ascribed to Carlocebus have been found, which provides some notion of the existent intra-specific variability of this species [37]. These tali have been described as similar to the Callitrichinae or to *Saimiri*, given their very broad, slightly medially directed talar neck, a broad shallow posterior calcaneal facet, and moderately low and broad trochlea [37]. *Carlocebus* has been usually interpreted in locomotor terms as exhibiting some moderate leaping and clambering, but mostly quadrupedal activities [38]. Our analyses of the biomechanical and morphometric classify this specimen as an arboreal quadruped (Table 4). The stress results obtained for *Carlocebus* are similar to *P. neuquenensis*, and to those of extant taxa such as *P. pithecia,* some *Callicebus* species, and *A. caraya* (which is the clamber/suspensory individual exhibiting

the lowest stress values) (Fig. 4). *C. carmenensis* is located in Fig. 8b. near *Aotus* and *Cebus apella*, which are well-known arboreal quadrupeds.

***Cebupithecia* sarmientoi**: This fossil species is well represented in La Venta, Colombia. It has been suggested that *C. sarmientoi* should be classified within Pitheciinae [39]. Nonetheless, *Cebupithecia* does not exhibit many Pitheciinae apomorphic characters in its post-cranium [38]. *Cebupithecia* has been usually considered as a mainly quadrupedal species [40] with moderate amounts of leaping, similar to *Callicebus* or the cebines [41]. The classification results obtained here categorised *Cebupithecia* as an arboreal quadruped, but the classification of the biomechanical also indicates leaping (Table 4). The stress results of *C. sarmientoi* are similar to the ones observed in *S. boliviensis* and *S. mystax* (which is the leaper exhibiting highest stress values, similar to the ones observed in mainly quadrupedal species) (Fig. 4). *C. sarmientoi* is positioned near *A. nancymaae* and *C. moloch* in the PCA of the morphometric data (Fig. 8b), which are also primarily quadrupedal species.

Dolichocebus gaimanensis: The oldest platyrrhine fossil with well-described postcranial elements is *D. gaimanensis* from the Sarmiento Formation, Chubut Province, Argentina [42]. The only postcranial element that has been ascribed to *D. gaimanensis* is the talus that was analysed in the present paper. It has been described as similar to medium-sized platyrrhines such *Callicebus, Cebus* and *Saimiri.* Its morphology has been interpreted as indicative of a generalised function with a predominance of frequent arboreal quadrupedal activities [43]. Our analyses show that *D. gaimanensis* is consistently and confidently classified

as an arboreal quadruped when analysing both the biomechanical and morphometric data (Table 4). *D. gaimanensis* shows stress values similar the fossil *N. fieldsi* or to those of the extant species *P. monachus*, *C. olivaceus, C. albifrons* and *A. azarae* (Fig. 4.). All these species are frequently interpreted as arboreal quadrupeds, excepting *Pithecia* that also shows some suspensory behaviours [44]. The PCA of the morphometric data (Fig. 8b) located *D. gaimanensis* near *C. albifrons* and *C. cupreus*, which are also species usually interpreted as predominately quadrupedal [40].

Madre de Dios: The Madre de Dios talus was found in the Peruvian Amazonia and it represents the first early record of a Miocene platyrrhine in northern South America [45]. The talus has not been taxonomically assigned yet. However, it has been depicted as showing a combination of different talar traits, which are mainly observable in the Cebinae [45]. Nevertheless, what is particularly outstanding about this fossil specimen is its small size, similar to marmosets and tamarins. Consequently, the Madre de Dios talus has been portrayed as a tiny Saimiri-like cebine primarily involved in arboreal quadrupedal activities, but also showing variable amounts of leaping and vertical clinging [45]. The present results classify Madre de Dios as an arboreal quadruped (Table 4), although the biomechanical data also indicates some leaping. The stress results of this specimen are similar to *S. fuscicollis*, *A. azarae* and *C. olivaceus.* In the PCA of the morphometric data (Fig. 8b) Madre de Dios occupies a position close to Río Cisnes, but not occupied by any other fossil or extant platyrrhine [40].

Neosaimiri fieldsi: Several skeletal elements of *N. fieldsi* have been discovered at La Venta, Colombia. The talar morphology of this species has been described to be similar to Saimiri, thus suggesting arboreal quadrupedalism as its main locomotor behaviour [40], although *N. fieldsi* possibly engaged in leaping with relative frequency as well [34,37]. The classification results here presented classify this fossil as a predominantly arboreal quadruped, although the analysis of the biomechanical data also suggests some leaping (Table 4). The stress results of *N. fieldsi* are similar to *C. olivaceus* or *C. albifrons* and to fossil specimens such as Madre de Dios and *D. gaimanensis* (Fig. 4). *Neosaimiri* is located in the morphometric PCA close to *C. torquatus* and near the species located at the negative extreme of PC2 (Fig. 8b).

Paralouatta marianae: The talus of *P. marianae* was found in the Early Miocene locality of Domo de Zaza, Cuba [46]. This talus is similar to *Paralouatta varonai*, although *P. marianae* is significantly smaller [47]. It has been argued that *Paralouatta* is quite distinct as compared to the extant platyrrhines [48], because it shows some traits (e.g., a noticeable cotylar fossa) that have been hypothesised to be related to locomotor behaviours such as terrestriallity or semi-terrestriality [47]. However, our analyses did not consider these categories so it is not possible to exclude these potential locomotor behaviours. The obtained classification results for *P. marianae* classified this specimen as either an arboreal quadruped or as a clamber/suspensory species (Table 4). In fact, the posterior probabilities for both the morphometric and biomechanical data are similar for both categories, so it is not possible to exclusively define one main locomotor category. These classification results are consistent with the stress

results observed in Figure 4, which are similar to the Atelidae. *P. marianae* is located in the morphometric PCA close to *Alouatta* and the most suspensory arboreal quadrupeds (i.e., *Pithecia*), thus indicating again clamber/suspensory traits.

Proteropithecia neuquenensis: *P. neuquenensis* corresponds to a medium-sized platyrrhine that is post-cranially represented by a single talus, which was found in the Collón Curá formation in Neuquén, Argentina [49]. *P. neuquenensis* has been usually classified as a pitheciin ancestor based on their teeth [49], nonetheless its talus has been described as similar to *Callicebus* or *Aotus* [37], and to fossil specimens such as Río Cisnes and *Proteropithecia* [40]. The talus has been depicted as exhibiting a wedged trochlea, moderate neck length, an oval head and an extended anterior proximal calcaneal face [49]. These traits have been interpreted as indicative of modedrate leaping and usual arboreal quadrupedalism [14]. Our results showed that *P. neuquenensis* was classified as an arboreal quadrupedalist (Table 8), although the biomechanical data also indicates leaping. The stress results of *P. neuquenensis* are similar to *P. pithecia* and *S. boliviensis* (Fig. 4), whilst in the PCA of the morphometric data this specimen is located near *C. donacophilus* (Fig. 8b).

Río Cisnes: The Río Cisnes talus from the Chilean site of Alto Río Cisnes represents the only post-cranial record of a primate in Chile. It is currently taxonomically unassigned and dates to the Friasian SALMA 16.4 Ma [50]. This talus is morphologically similar to *Callicebus* or a smaller *Carlocebus* [50]. It has been proposed that the moderately high talar body with the parallel-sided rims

and the relatively long neck could indicate some leaping in what otherwise seems to be a generalised arboreal quadruped [37,40,51]. The classification results obtained in the present paper indicate an arboreal quadruped, although the posterior probabilities for other locomotor behaviours are not totally negligible (Table 4). The stress values of Río Cisnes are comparable to those of *S. boliviensis*, *P. pithecia* or *S. mystax* (which is the leaper exhibiting highest stress values) (Fig. 4). The Río Cisnes talus is positioned close to Madre de Dios in the PCA of the morphometric data (Fig. 8b), which as previously explained corresponds to an area of the morphospace not occupied by any other specimen under analysis, perhaps representing some unique locomotor adaptations that will require further analysis.

Soriacebus ameghinorum: *Soriacebus* was also found in the Pinturas formation and was originally portrayed as being similar to Pitheciinae or the Callitrichinae [52]. However, it was later categorised as an early member of the Pitheciinae [53] or as a stem platyrrhine [54]. The single available talus that was analysed in the present work has been described as similar to those of *Alouatta* and *Pithecia* [37]. The present analysis classifies this fossil specimen as an arboreal quadruped (Table 4), with special emphasis when analysing the morphometric data. The stress results of *Soriacebus* are similar to the ones observed in *Cacajao calvus* and *Cebus apella* (Fig. 4), whereas the in the PCA (Fig. 8b) this fossil individual is close to *Chiropotes satanas.*

References

1. Hébert D, Lebrun R, Marivaux L. 2012 Comparative three-dimensional structure of the trabecular bone in the talus of primates and its relationship to ankle joint loads generated during locomotion. *Anat. Rec. Adv. Integr. Anat. Evol. Biol.* **295**, 2069–2088. (doi:10.1002/ar.22608)

2. Zollikofer CP, Leon MP de. 2005 *Virtual reconstruction: A primer in computer-assisted paleontology and biomedicine*. 1 edition. Hoboken, N.J: Wiley-Liss.

3. Gunz P, Mitteroecker P, Neubauer S, Weber GW, Bookstein FL. 2009 Principles for the virtual reconstruction of hominin crania. *J. Hum. Evol.* **57**, 48–62. (doi:10.1016/j.jhevol.2009.04.004)

4. Weber GW, Bookstein FL. 2011 *Virtual anthropology: a guide to a new interdisciplinary field*. Wien; London: Springer.

5. Püschel TA, Gladman JT, Bobe R, Sellers WI. 2017 The evolution of the platyrrhine talus: A comparative analysis of the phenetic affinities of the Miocene platyrrhines with their modern relatives. *J. Hum. Evol.* **111**, 179–201. (doi:10.1016/j.jhevol.2017.07.015)

6. Wiley DF *et al.* 2005 Evolutionary morphing. In *EEE Visualization, 2005. VIS 05*.

7. Marcé-Nogué J, De Esteban-Trivigno S, Escrig C, Gil L. 2016 Accounting for differences in element size and homogeneity when comparing Finite Element models: Armadillos as a case study. *Palaeontol. Electron.* **19**, 1–22.

8. Marcé-Nogué J, Fortuny J, Gil L, Sánchez M. 2015 Improving mesh generation in Finite Element Analysis for functional morphology approaches. *Span. J. Palaeontol.* **31**, 117–132.

9. Walmsley CW, Smits PD, Quayle MR, McCurry MR, Richards HS, Oldfield CC, Wroe S, Clausen PD, McHenry CR. 2013 Why the long face? The mechanics of mandibular symphysis proportions in crocodiles. *PLoS ONE* **8**, e53873. (doi:10.1371/journal.pone.0053873)

10. Marcé-Nogué J, Esteban-Trivigno SD, Püschel TA, Fortuny J. 2017 The intervals method: a new approach to analyse finite element outputs using multivariate statistics. *PeerJ* **5**, e3793. (doi:10.7717/peerj.3793)

11. Kuhn M, Johnson K. 2013 *Applied Predictive Modeling*. 2013 edition. New York: Springer.

12. Hastie T, Tibshirani R, Friedman J. 2017. *The elements of statistical learning: Data mining, inference, and prediction.* 2nd edn. New York, NY: Springer.

13. Kotsiantis SB, Zaharakis I, Pintelas P. 2007 Supervised machine learning: A review of classification techniques. *Emerg. Artif. Intell. Appl. Comput. Eng.* **160**, 3–24.

14. Alpaydin E. 2016 *Machine Learning: The new AI*. Cambridge, MA: MIT Press.

15. Kuhn M. 2008 Caret package. *J. Stat. Softw.* **28**, 1–26.

16. Fisher RA. 1936 The use of multiple measurements in taxonomic problems. *Ann. Hum. Genet.* **7**, 179–188.

17. Welch BL. 1939 Note on discriminant functions. *Biometrika* **31**, 218–220.

18. Venables WN, Ripley BD. 2013 *Modern applied statistics with S-PLUS*. Springer Science & Business Media.

19. Peterson LE. 2009 K-nearest neighbor. *Scholarpedia* **4**, 1883. (doi:10.4249/scholarpedia.1883)

20. Hsu C-W, Chang C-C, Lin C-J. 2003 A practical guide to support vector classification. Tech. rep. Department of Computer Science, National Taiwan University.

21. Vapnik V. 2000 *The nature of statistical learning Theory*. 2nd edn. New York: Springer-Verlag.

22. Decoste D, Schölkopf B. 2002 Training invariant support vector machines. *Mach. Learn.* **46**, 161–190. (doi:10.1023/A:1012454411458)

23. Ben-Hur A, Ong CS, Sonnenburg S, Schölkopf B, Rätsch G. 2008 Support vector machines and kernels for computational biology. *PLOS Comput. Biol.* **4**, e1000173. (doi:10.1371/journal.pcbi.1000173)

24. Schölkopf B, Smola AJ. 2002 *Learning with kernels: support vector machines, regularization, optimization, and beyond*. MIT press.

25. Zhang H. 2004 The optimality of naive Bayes. *AA* **1**, 3.

26. Webb GI, Boughton JR, Wang Z. 2005 Not so naive Bayes: Aggregating one-dependence estimators. *Mach. Learn.* **58**, 5–24. (doi:10.1007/s10994-005-4258-6)

27. Rennie JD, Shih L, Teevan J, Karger DR. 2003 Tackling the poor assumptions of naive bayes text classifiers. In *Proceedings of the 20th international conference on machine learning (icml-03)*, pp. 616–623.

28. Caruana R, Niculescu-Mizil A. 2006 An empirical comparison of supervised learning algorithms. In *Proceedings of the 23rd international conference on Machine learning*, pp. 161–168. ACM.

29. Loh W-Y. 2011 Classification and regression trees. *Wiley Interdiscip. Rev. Data Min. Knowl. Discov.* **1**, 14–23. (doi:10.1002/widm.8)

30. Breiman, L. 1984. Classification and regression trees. New York: Routledge.

31. Lin Y, Jeon Y. 2002 Random forests and adaptive nearest neighbors. *J. Am. Stat. Assoc.* , 101–474.

32. Ho TK. 1995 Random decision forests. In *Document analysis and recognition, 1995., proceedings of the third international conference on*, pp. 278–282. IEEE.

33. Setoguchi T, Rosenberger AL. 1987 A fossil owl monkey from La Venta, Colombia. *Nature* **326**, 692–694. (doi:10.1038/326692a0)

34. Daniel L. Gebo MD. 1990 New primate tali from La Venta, Colombia. *J. Hum. Evol.* **19**, 737–746. (doi:10.1016/0047-2484(90)90005-V)

35. Gebo DL. 1988 Foot morphology and locomotor adaptation in Eocene primates. *Folia Primatol. Int. J. Primatol.* **50**, 3–41.

36. Tejedor MF. 2005 New specimens of Soriacebus adrianae Fleagle, 1990, with comments on pitheciin primates from the Miocene of Patagonia. *Ameghiniana* **42**, 249–251.

37. Meldrum DJ. 1990 New fossil platyrrhine tali from the early miocene of Argentina. *Am. J. Phys. Anthropol.* **83**, 403–418. (doi:10.1002/ajpa.1330830402)

38. Ford SM. 1990 Locomotor adaptations of fossil platyrrhines. *J. Hum. Evol.* **19**, 141–173. (doi:10.1016/0047-2484(90)90015-4)

39. Hartwig WC, Meldrum DJ. 2002 Miocene platyrrhines of the northern Neotropics. *Camb. Stud. Biol. Evol. Anthropol.* , 175–188.

40. Püschel TA, Gladman JT, Bobe R, Sellers WI. 2017 The evolution of the platyrrhine talus: A comparative analysis of the phenetic affinities of the Miocene platyrrhines with their modern relatives. *J. Hum. Evol.* **111**, 179–201. (doi:10.1016/j.jhevol.2017.07.015)

41. Meldrum DJ, Lemelin P. 1991 Axial skeleton of Cebupithecia sarmientoi (Pitheciinae, Platyrrhini) from the middle miocene of La Venta, Colombia. *Am. J. Primatol.* **25**, 69–89. (doi:10.1002/ajp.1350250202)

42. Kay RF, Fleagle JG, Mitchell TRT, Colbert M, Bown T, Powers DW. 2008 The anatomy of Dolichocebus gaimanensis, a stem platyrrhine monkey from Argentina. *J. Hum. Evol.* **54**, 323–382. (doi:10.1016/j.jhevol.2007.09.002)

43. Meldrum DJ. 1993 Postcranial adaptations and positional behavior in fossil platyrrhines. *Postcranial Adapt. Nonhum. Primates North. Ill. Univ. DeKalb* , 235–251.

44. Youlatos D, Meldrum J. 2011 Locomotor Diversification in New World Monkeys: Running, Climbing, or Clawing Along Evolutionary Branches. *Anat. Rec. Adv. Integr. Anat. Evol. Biol.* **294**, 1991–2012. (doi:10.1002/ar.21508)

45. Marivaux L, Salas-Gismondi R, Tejada J, Billet G, Louterbach M, Vink J, Bailleul J, Roddaz M, Antoine P-O. 2012 A platyrrhine talus from the early Miocene of Peru (Amazonian Madre de Dios Sub-Andean Zone). *J. Hum. Evol.* **63**, 696–703. (doi:10.1016/j.jhevol.2012.07.005)

46. MacPhee RDE, Iturralde-Vinent M, Gaffney ES. 2003 *Domo de Zaza, an Early Miocene vertebrate locality in South-Central Cuba: With notes on the tectonic evolution of Puerto Rico and the Mona Passage*. American Museum of Natural History.

47. MacPhee RDE, Meldrum J. 2006 Postcranial remains of the extinct monkeys of the Greater Antilles, with evidence for semiterrestriality in Paralouatta. *Am. Mus. Novit.* **16**. (doi:10.1206/0003-0082(2006)3516[1:PROTEM]2.0.CO;2)

48. MacPhee RDE, Iturralde-Vinent MA. 1995 Earliest monkey from Greater Antilles. *J. Hum. Evol.* **28**, 197–200. (doi:10.1006/jhev.1995.1014)

49. Kay RF, Johnson D, Meldrum DJ. 1998 A new pitheciin primate from the middle Miocene of Argentina. *Am. J. Primatol.* **45**, 317–336. (doi:10.1002/(SICI)1098-2345(1998)45:4<317::AID-AJP1>3.0.CO;2-Z)

50. Tejedor MF. 2003 New fossil primate from Chile. *J. Hum. Evol.* **44**, 515–520. (doi:10.1016/S0047-2484(03)00026-5)

51. Gebo DL, Simons EL. 1987 Morphology and locomotor adaptations of the foot in early Oligocene anthropoids. *Am. J. Phys. Anthropol.* **74**, 83–101. (doi:10.1002/ajpa.1330740108)

52. Luchterhand K, Kay RF, Madden RH. 1986 Mohanamico hershkovitzi, gen. et sp. Nov., un primate du Miocène moyen d'Amérique du Sud. *Comptes Rendus Académie Sci. Paris* **Ser. II**, **303**, 1753–1758.

53. Tejedor MF. 2008 The origin and evolution of Neotropical Primates. *Arq. Mus. Nac. Rio Jan.* **66**, 251–269.

54. Kay RF, Fleagle JG. 2010 Stem taxa, homoplasy, long lineages, and the phylogenetic position of Dolichocebus. *J. Hum. Evol.* **59**, 218–222.
