# Supplementary material for: Inferring locomotor behaviours in Miocene New World monkeys using finite element analysis, geometric morphometrics and machine-learning classification techniques applied to talar morphology
Source: J R Soc Interface. 2018 Sep 26;15(146):20180520. doi: 10.1098/rsif.2018.0520 (PMC6170775; doi:10.1098/rsif.2018.0520)
Supplement: Phylomorphospace of the extant platyrrhine sample computed using the morphometric data. [file rsif20180520supp6.pdf]

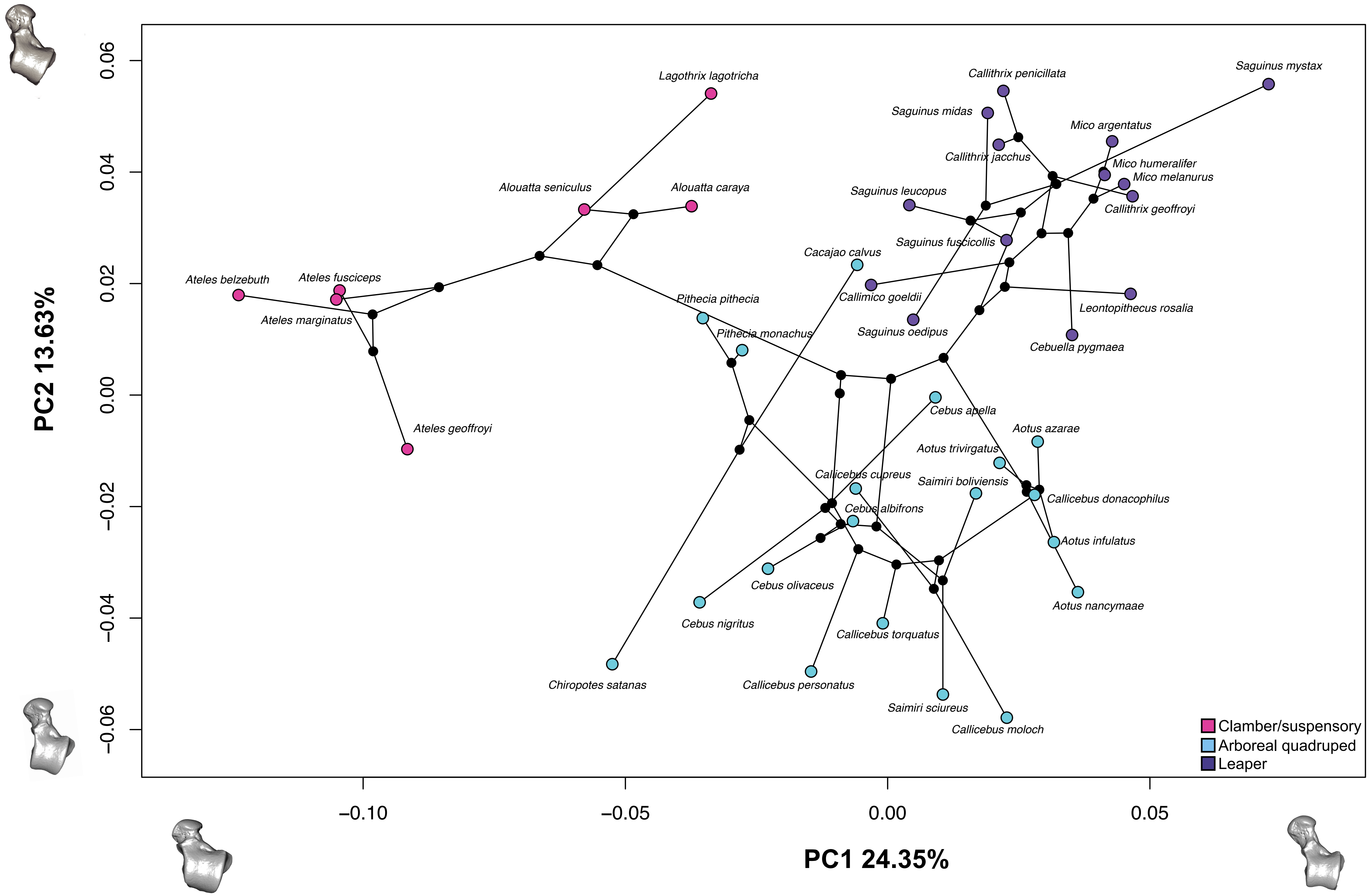

S6. Phylomorphospace of the extant platyrrhine sample (only the first two PCs are shown). One of the models closest to the mean shape was warped to match the multivariate mean using the thin plate spline method, then the obtained average model was warped to represent the variation along the two plotted PC axes in both analyses. The black dots correspond to the nodes of the projected phylogeny.
